# Human Action Recognition Using Improved Salient Dense Trajectories

**DOI:** 10.1155/2016/6750459

**Published:** 2016-05-17

**Authors:** Qingwu Li, Haisu Cheng, Yan Zhou, Guanying Huo

**Affiliations:** Key Laboratory of Sensor Networks and Environmental Sensing, Hohai University, Changzhou 213022, China

## Abstract

Human action recognition in videos is a topic of active research in computer vision. Dense trajectory (DT) features were shown to be efficient for representing videos in state-of-the-art approaches. In this paper, we present a more effective approach of video representation using improved salient dense trajectories: first, detecting the motion salient region and extracting the dense trajectories by tracking interest points in each spatial scale separately and then refining the dense trajectories via the analysis of the motion saliency. Then, we compute several descriptors (i.e., trajectory displacement, HOG, HOF, and MBH) in the spatiotemporal volume aligned with the trajectories. Finally, in order to represent the videos better, we optimize the framework of bag-of-words according to the motion salient intensity distribution and the idea of sparse coefficient reconstruction. Our architecture is trained and evaluated on the four standard video actions datasets of KTH, UCF sports, HMDB51, and UCF50, and the experimental results show that our approach performs competitively comparing with the state-of-the-art results.

## 1. Introduction

Human action recognition is an important research direction in computer vision field because of the requirement of real-world application, such as video indexing, video surveillance, and human-computer interaction [1–3]. Although a large number of human action recognition algorithms have emerged in recent years, the existing human action recognition features are still redundant and defective due to the high complexity, variability, background interference, and other factors of the human body movement. Therefore, human action recognition is still a hot and difficult problem in computer vision field. Since the human actions have high complexity and variability, the human action recognition model based on templates needs large numbers of human action templates prototypes and it will take a heavy price in the storage and transmission. Thus, it is critical to build a robust action representation for further recognition.

To achieve this goal, many researchers have worked on approaches with different motivations [4–6]. Some approaches have been proposed to reduce the features dimensions. For example, Laptev and Lindeberg [7] propose a method which uses the space time interest point detector approach to find the unique spatiotemporal features similar to finding Harris corners in images. Some approaches are devoted to discover the correlation between scene and human actions. Marszałek et al. [8] introduce the context relationship of human actions and natural dynamic scenes to improve the human action recognition accuracy. And several works are made to alleviate the effect of unwanted background local features on video representation. Wang et al. [9] introduce a descriptor based on motion boundary histograms (MBH) which rely on differential optical flow to improve the dense trajectories features. Liu et al. [10] describe a feature selection approach which uses motion statistics to acquire stable motion features and cleans static features for better training and recognition. Chakraborty et al. [11] propose a selective spatiotemporal interest point detector which applies surrounding suppression combined with local and temporal constraints. Besides, some works have proposed novel structures to improve the recognition accuracy. Chen et al. [12] propose a new spatiotemporal interest point detector based on flow vorticity which can not only suppress most of effects of camera motion but also provide prominent spatiotemporal interest points around key positions of the moving foreground. O'Hara and Draper [13] present a novel structure designed to provide an efficient approximate nearest-neighbor query of subspaces represented as points on Grassmann manifolds. Liu et al. [14] sample the motion salient region via the energy function and make the interest points distributed in the motion intense region. Qin et al. [15] have proposed a novel approach based on composite spatiotemporal features, which combines 3D histograms of oriented gradients feature with histograms of optical flow feature. Wang et al. [16] have proposed more effective dense trajectories for describing videos inspired by the recent success of dense sampling in image classification, namely, sampling dense points from each frame, and tracking them based on displacement information from a dense optical flow field.

**Figure 1:**
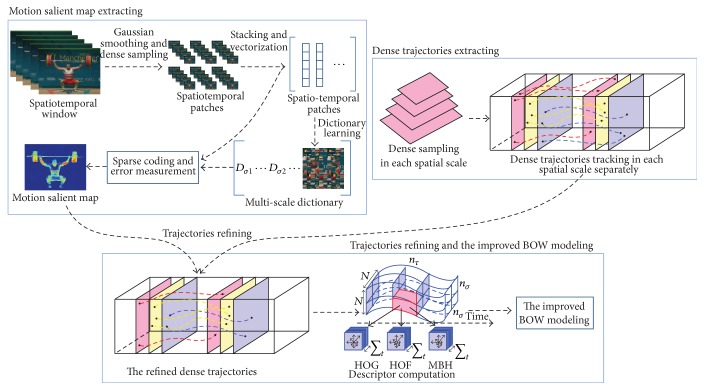
The main architecture of the improved human action recognition algorithm.

Among all the existing human action recognition algorithms, the trajectory-based technology is one of the newest research hotspots [17]. However, there exist two key points while utilizing the trajectory-based technology in human action recognition: the refinement of trajectories and the effectiveness of feature representation. Wang et al. [16] refine the trajectories only from the aspect of geometry; thus, the removed feature points are not always unimportant. This paper proposes a novel method that refines the dense trajectories via the analysis of the motion saliency of current frame and adjacent frame from the aspect of biology and optimizes the framework of bag-of-words according to the motion salient intensity distribution and the idea of sparse coefficient reconstruction. The main architecture of the improved human action recognition algorithm proposed in this paper is shown in Figure 1.

The rest of this paper is organized as follows.  Section 2 briefly introduces the core theory and method to be utilized.  Section 3 presents a detailed method about the improved algorithm (i.e., the improved dense trajectory features and the improved BOW approach).  Section 4 dwells on the overall evaluations and discussions on the algorithm.  Section 5 draws conclusions and proposes future work.

## 2. Theory and Method

### 2.1. Motion Saliency Detection

The method of spatiotemporal interest points has been applied in human action recognition successfully, but it often contains many interest points which are not relevant to human actions and affect the final recognition accuracy seriously, such as the interest points lying in the complex background. Therefore, it is very important to refine the interest points before feature extraction, and the analysis of the motion saliency is an effective and common method.

Somasundaram et al. [18] draw the motivation from the theory of Kolmogorov complexity and entropy and determine the most informative spatiotemporal regions in a video sequence as defined by its description length. The more complex (or longer) the description length of a spatiotemporal patch, the more informative or complex the patch. They propose the method that uses the sparse representation error to represent the motion saliency value of each spatiotemporal patch.

For a *b* × *b* × *w* spatiotemporal patch in a video, it can be vectorized to form a data vector **x**. Assuming the data can be encoded by a given basis dictionary *D* and a coefficient vector *α*, the reconstruction error follows a Gaussian distribution: *p*(*x*∣*D*, *α*) ~ *N*(*Dα*, *β* ^2^), where *β* is a standard deviation. Further, assuming parsimony of data representation, only a few dimensions in the coefficient vector are assumed to be used. Referring to the approximate solution of Kolmogorov complexity, the idea of minimum description length (MDL), the data vectors are assumed to be representable by a few columns of *D*, while the noise part cannot be represented by any combination of the columns. Incorporating these assumptions and writing *α* explicitly in terms of its sparsity *k* lead to

(1) α^≔minα1β2x−Dα22,α0≤k,

where ‖*α*‖_0_ stands for the number of nonzero dimensions in *α* and *k* controls the sparsity of **x** in *D*. ‖*α*‖_0_ is defined as the description length of the data vector **x** and minimizes the objective in (1). For formula (1), there exist two main problems: (1) it is a nonconvex combinatorial problem and is known to be NP-hard and (2) the dictionary is assumed to be given.

Assuming that the dictionary *D* ∈ *ℜ* ^*d*×*n*^ has *d*_1_, *d*_2_,…, *d*_*n*_ as its columns, there exists the following standard dictionary learning and sparse coding problem variant of (1):

(2) $$\begin{array}{c} \underset{\alpha ,D}{min}\overset{N}{\underset{i=1}{\sum }}{\left\|\;x_{i}-D\alpha_{i}\;\right\|}_{2}^{2}+\lambda {\left\|\;\alpha_{i}\;\right\|}_{1},\\ {\left\|\;d_{j}\;\right\|}_{2}\leq 1,\;j=1,2,\ldots ,n, \end{array}$$

where **x**_*i*_, *i* = 1,2,…, *N*, represents the data vectors and *λ* is a regularization constant. The inequality constraints on the dictionary atoms are added to avoid degenerate cases while learning the dictionary. For formula (2), the dictionary *D* can be solved using the *K*-SVD algorithm, and the sparse representation coefficient *α*(**x**, *D*) can be solved quickly using orthogonal matching pursuit (OMP); then, the sparse representation residual of the data vector **x** is given by

(3) $$\begin{array}{c} R\left(\;x,D,\alpha \;\right)={\left\|\;x-D\alpha \left(\;x,D\;\right)\;\right\|}_{2}. \end{array}$$

### 2.2. Dense Trajectories

The trajectory-based human action feature representation has proved very effective. Typically, the trajectories are extracted by KLT tracking [19] or SIFT matching between frames [20], but the quantity and quality of these trajectories are often insufficient. Wang et al. [16] propose the more effective dense trajectories for describing videos inspired by the recent success of dense sampling in image classification. For each frame in videos, feature points are sampled on a grid spaced by *W* pixels and tracked in each scale separately. Experimental results show that the sampling step size of *W* = 5 is dense enough to give good results. According to the resolution of videos, each frame is set to 8 spatial scales by a factor $1/\sqrt{2}$. The dense optical flow field between frame *t* and the next frame *t* + 1 is *w*_*t*_ = (*u*_*t*_, *v*_*t*_), where *u*_*t*_ is the vertical component and *v*_*t*_ is the horizontal component. For the feature point *P*_*t*_ = (*x*_*t*_, *y*_*t*_) at frame *t*, it can be tracked to *P*_*t*+1_ = (*x*_*t*+1_, *y*_*t*+1_) at the next frame *t* + 1 by median filtering in the dense optical flow field *w*_*t*_, and the location of *P*_*t*+1_ is defined as follows:

(4) $$\begin{array}{c} P_{t+1}=\left(\;x_{t+1},y_{t+1}\;\right)=\left(\;x_{t},y_{t}\;\right)+{\left.\;\left(\;M*w_{t}\;\right)\;\right|}_{\left(x,y\right)}, \end{array}$$

where *M* is the 3 × 3 median filtering kernel. Points of subsequent frames are concatenated to form a trajectory: (*P*_*t*_, *P*_*t*+1_, *P*_*t*+2_,…), but there exists a very common problem in tracking: drifting. In order to avoid this circumstance as much as possible, the length of a trajectory is limited to *L*. For each frame, if the tracking point is not found in a *W* × *W* neighborhood, then we need to resample a feature and add it to the tracking process. Giving a trajectory of length *L*, the motion shape can be represented by the sequence *S* = (Δ*P*_*t*_,…, Δ*P*_*t*+*L*−1_) of displacement vectors Δ*P*_*t*_ = (*P*_*t*+1_ − *P*_*t*_) = (*x*_*t*+1_ − *x*_*t*_, *y*_*t*+1_ − *y*_*t*_). Then, the normalization of the motion trajectory shape is

(5) $$\begin{array}{c} S^{\prime }=\frac{\left(\Delta P_{t},\ldots ,\Delta P_{t+L-1}\right)}{\sum_{j=t}^{t+L-1}\left\|\Delta P_{j}\right\|}, \end{array}$$

where the normalization factor is the sum of the magnitudes of the displacement vectors.

## 3. Feature Extraction and Representation

### 3.1. Improved Dense Trajectories

In the research field of human action recognition algorithms, Wang and Zhao [21] proved that refining the features can improve the recognition accuracy effectively. The dense trajectory proposed by [16] has a good result, but the method of refining the trajectories is still not perfect. Wang et al. [16] consider that trajectories with little displacements are most likely to be background and meanwhile trajectories with large displacements are most likely to be drifting problem. These trajectories need to be removed. However, by refining the trajectories only from the aspect of geometry, the removed feature points are not always unimportant. This paper proposes a novel method from the aspect of biology that refines the dense trajectories via the analysis of the motion saliency of current frame and adjacent frame.

**Figure 2:**
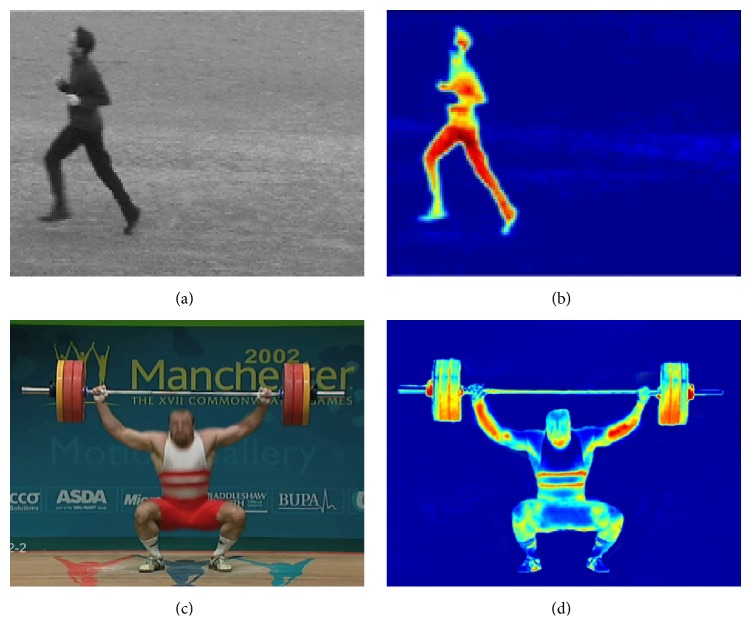
The effective images of the motion salient map. (a) One original frame of running videos in KTH dataset. (b) Motion salient map of running action. (c) One original frame of lifting videos in UCF sports dataset. (d) Motion salient map of lifting action.

We first extract the motion salient region in videos. According to the default parameters in [18], for each video sequence, it is made up of many sliding temporal windows, and then each temporal window is arranged in a 3-level Gaussian pyramid. Each pyramid level is densely sampled into spatiotemporal patches of size 3 × 3 × 2. The spatiotemporal patches are then vectorized to a matrix **x** and generated three scales matrix (including the native scale) according to the 3-level Gaussian pyramid. Then, we can learn the corresponding dictionary separately from each level, and these dictionaries are then concatenated column-wise into a single multiscale dictionary. The sparse representation coefficient of each spatiotemporal patch can be done by OMP; therefore, we can calculate the sparse representation residual of each frame and normalize it as the motion salient map *S*_*t*_(*x*, *y*). The effective images of motion salient extraction are shown in Figure 2.

Then, we use the method proposed in [16] to get the original trajectories *T*_1_ = {*P*_(*t*,*i*)_}; *P*_(*t*,*i*)_ is the location of the *i*th feature point in frame *t*. In the motion salient map, we define the area as the background or unimportant feature points where the intensity of the feature points is less than *λ*_1_ and define some messy trajectories as the drifting problem that the intensity difference of the feature points between frames is greater than *λ*_2_. These two types of trajectories are the disturbance and should be removed; therefore, the refined trajectories in this paper is defined as follows:

(6) T2=Pt,i ∣ Pt,i∈T1,  StPt,i≥λ1,  StPt,i−St+1Pt+1,i≤λ2.

Set the trajectory length *L* = 15; then, the corresponding trajectory displacement vector and the normalization trajectory displacement are as follows:

(7) ΔT2=ΔPt,i=Pt,i−Pt+1,i ∣ Pt,i∈T2,T2′=ΔT2∑j=tt+L−1ΔPj.

**Figure 3:**
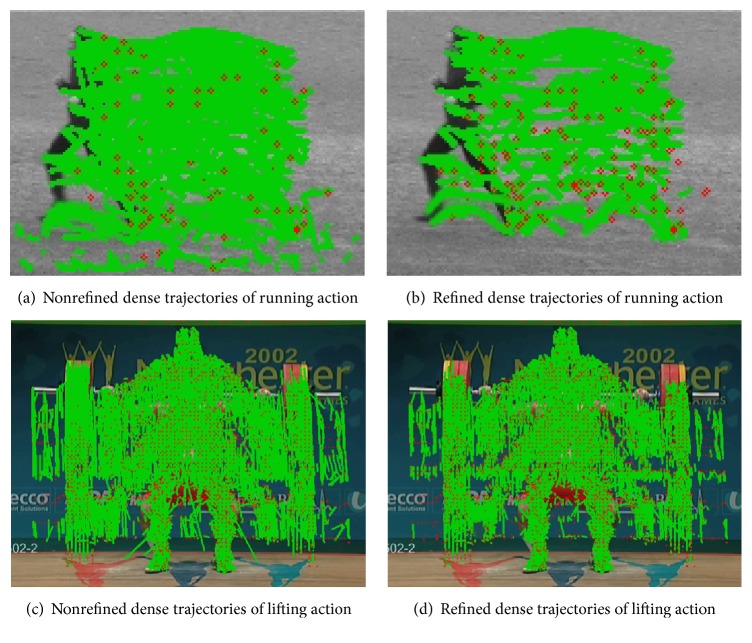
Examples of nonrefined and refined dense trajectories in this paper.

**Figure 4:**
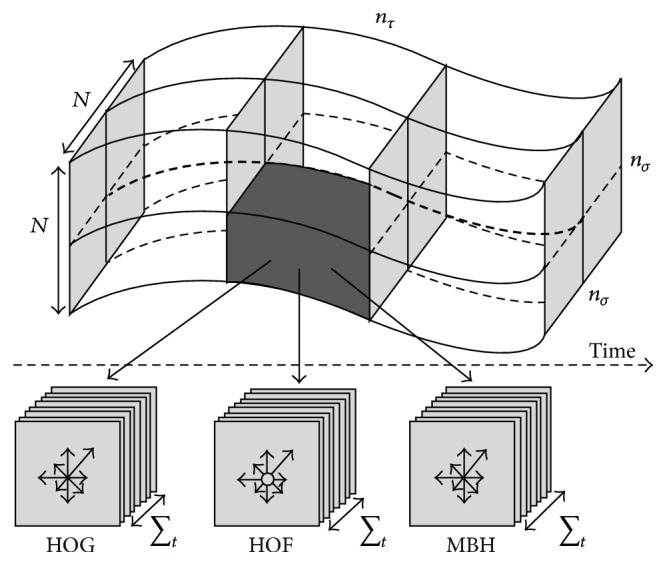
Illustration of multifeature computation.

Figure 3 shows the nonrefined and refined dense trajectories in this paper.  Figure 3(a) shows the nonrefined dense trajectory of the running action in KTH dataset and Figure 3(b) shows the refined dense trajectory of the running action in KTH dataset.  Figure 3(c) shows the nonrefined dense trajectory of the lifting action in UCF sports dataset.  Figure 3(d) shows the refined dense trajectory of the lifting action in UCF sports dataset. We can observe that some drifted trajectories in Figures 3(a) and 3(c) are removed as shown in Figures 3(b) and 3(d). Besides the trajectory displacement features, the trajectory-based HOG/HOF and MBH features are also constructed to represent the appearance and motion information of human actions. HOG focuses on static appearance information, whereas HOF and MBH capture the local motion information. As depicted in Figure 4, for each trajectory, we use the default parameters in [16], set the spatiotemporal size *N* × *N* × *L*, where *N* = 32 and *L* = 15, and calculate the feature descriptors in the spatiotemporal patches aligned with the trajectories according to the motion information of the dense trajectories.

### 3.2. Improved BOW Approach

**Figure 5:**
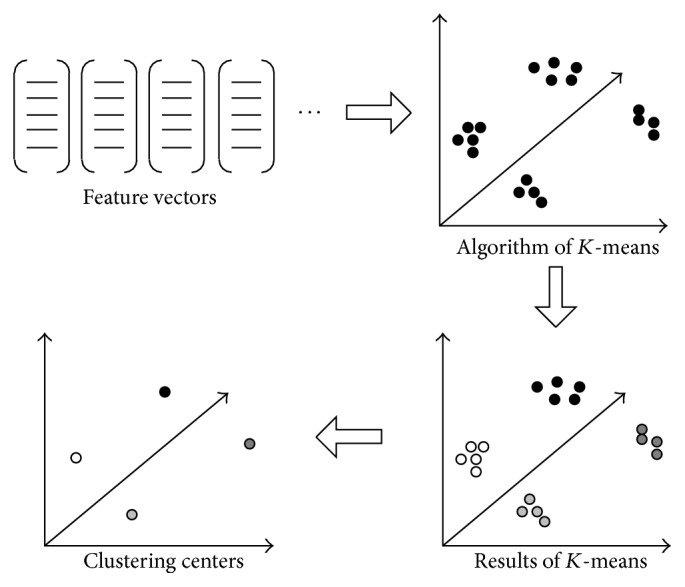
The flow of constructing the dictionary.

**Figure 6:**
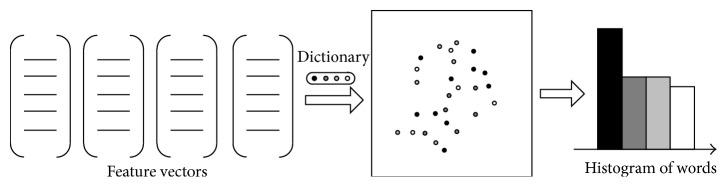
The generation of histogram of words.

In recent years, bag-of-words (BOW) approach [22–24] is often adopted in human action recognition algorithms based on spatiotemporal features. Each column feature vector is represented by the closest word in the visual dictionary; then, the representation of each video can be converted from large numbers of feature vector descriptors to a words frequency histogram. Most of the visual dictionary construction methods are applying the traditional *K*-means clustering; it mainly contains three steps: (1) The extraction of feature vectors: HOG (or HOF) is implemented to translate an image into feature vectors. (2) Constructing the dictionary by *K*-means: The central idea of *K*-means clustering is to minimize distance within the class, and the sample data will be divided into *K* classes scheduled. The flow of *K*-means is as follows: (a) Choose *K* feature vectors randomly as the initial clustering center. (b) Calculate the distance between all the feature vectors and clustering center and choose the nearest class as the class of the feature vector. (c) Calculate the mean value of each class. (d) Execute (b) and (c) until clustering is unchanged. Thus, the *K* clustering centers construct the dictionary. The flow of constructing the dictionary is shown in Figure 5. (3) Represent the image by the histogram of visual words: Each feature vector can be approximately replaced by the words of dictionary; then, make a count on each word to generate the histogram of words, as illustrated in Figure 6.

However, the visual dictionary constructed is very rough due to the tight limits of the traditional *K*-means clustering, and the feature vector is distributed to the nearest visual word rigidly. Actually, the central distances between the feature vector and the visual words, namely, the contributions to the visual words, are not equal completely, and they all have different weights. Motion salient map can not only highlight the human body movement region but also measure the movement intensity of human body's each part. Therefore, the motion salient map can be used to train a more accurate visual dictionary, and the idea of sparse coding can be used to get a better video representation. In this paper, we propose a method that optimizes the construction of the visual dictionary in the framework of BOW according to the motion salient intensity distribution and the idea of sparse coding.

In all the training videos, assume that the set of all the trajectories is *T* = {*T*_*i*_} and the corresponding set of the feature descriptors is *X* = {*x*_*i*_}. The weights of all the trajectories can be calculated according to the motion salient intensity distribution:

(8) $$\begin{array}{c} w_{i}=\overset{t+L-1}{\underset{n=t}{\sum }}\frac{s_{n}\left(P_{\left(n,m\right)}\right)}{L}, \end{array}$$

where *m* is the feature point number in the trajectory *T*_*i*_. Then, we use weighted *K*-means algorithm to construct the optimal visual dictionary:

(9) arg minX ∑j=1k ∑xi∈Xwixi−zj2,

where *Z* = {*z*_*j*_} is the optimal visual dictionary.

In the framework of BOW method, each action video is represented as a *k* long vector, *H* = [*h*_1_,…, *h*_*k*_], namely, words frequency histogram, where *h*_*j*_ is the number of times the nearest neighbor of a descriptor in the video is found to be *z*_*j*_. Given that the video descriptors *X* = {*x*_*i*_} have the corresponding weight *w*_*i*_, we modify the Euclidean distance formula:

(10) $$\begin{array}{c} Distance_{ij}=w_{i}{\left\|\;x_{i}-z_{j}\;\right\|}^{2}. \end{array}$$

However, *K*-means algorithm is too strict on classification and resulting in the limited description of the feature vector *x*_*i*_; namely, it is a little rough on the construction of the original feature vector. In order to alleviate this problem, we use the idea of the coefficient reconstruction of sparse coding to optimize the coefficient of *x*_*i*_ in the visual dictionary *Z* = {*z*_*j*_} rather than directly classifying the descriptor vector *x*_*i*_ to the nearest word *z*_*j*_. The steps are as follows:

(1) Calculate all the distances *D*_*ij*_ between the feature vector *x*_*i*_ and each word in the visual dictionary *Z* = {*z*_*j*_}.
(2) Normalize all the distances to get the weighed coefficient *HW*_*ij*_.
(3) Calculate the words frequency histogram *H*′ = [*h*_1_′,…, *h*_*k*_′] according to the formula: *h*_*j*_′ = ∑_*i*_ *HW*_*ij*_.

## 4. Experiment Evaluations and Discussions

### 4.1. Datasets

**Figure 7:**
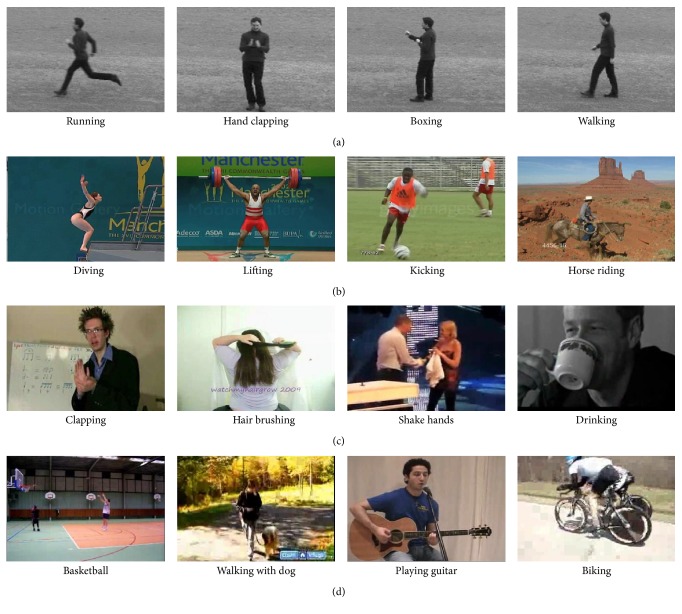
Illustration of some action sequences from the four datasets used in our experiment. ((a)–(d)) KTH, UCF sports, HMDB51, and UCF50.

In order to verify the effectiveness of the human action recognition algorithm in this paper, we will evaluate this algorithm on the two public datasets, including KTH, UCF sports, HMDB51, and UCF50 datasets (see Figure 7).

KTH dataset [25] is widely applied in the research experiment of human action recognition. This dataset contains six different types of action videos, including walking, jogging, running, boxing, hand waving, and hand clapping. These action videos are shooting in 4 different scenes (e.g., indoor, outdoor, outdoor camera scale change, and outdoor clothing change) by 25 volunteers. All the videos are shooting using the static camera, the frame rate is 25 fps, and the resolution ratio is 320 × 240. In addition, all the videos' backgrounds are homogeneous, and the average time length of videos is 4 s.

UCF sports dataset [26] contains 10 categories of sports derived from sports videos, including diving, kicking, lifting, horse riding, running, skateboarding, golf swing, swing bench, swing side angle, and walking. The dataset has a total of 150 videos and has different frame rates and different resolution ratios. In addition, these video claps are shooting in a variety of scenes, the objective scale and view angle change obviously, and the average time length of videos is 5 s.

The HMDB51 dataset [27] is collected from a variety of sources ranging from digitized movies to YouTube videos. It contains simple facial actions, general body movements, and human interactions. In total, there are 51 action categories and 6,766 video sequences. For every class and split, there are 70 videos for training and 30 videos for testing. Note that the dataset includes both the original videos and their stabilized version.

The UCF50 dataset [28] has 50 action categories, consisting of real-world videos taken from the YouTube website. This dataset can be considered as an extension of the YouTube dataset. The actions range from general sports to daily life exercises. For all 50 categories, the videos are split into 25 groups. For each group, there are at least 4 action clips. In total, there are 6,618 video clips. The video clips in the same group may share some common features, such as the same person, similar background, or similar viewpoint.

### 4.2. Developing Environment and Classifier

The algorithm proposed in this paper is executed in Linux, the developing environment is QT Creator + OpenCV, and the computer hardware is Intel® Core*™* i7-4700HQ CPU @ 2.4 GHZ 16 GB RAM.

For the classifier, we adopt the common nonlinear support vector machine (SVM) and choose the kernel function *χ* ^2^:

(11) $$\begin{array}{c} K\left(\;H_{i},H_{j}\;\right)=\exp\left(\;-\frac{1}{A}\underset{k}{\sum }\frac{{\left(H_{i,k}-H_{j,k}\right)}^{2}}{H_{i,k}+H_{j,k}}\;\right). \end{array}$$

### 4.3. Optimal Parameters Analysis

The main goal of the experiment is to verify the influence on the recognition accuracy by using the two aspects of the improved algorithms proposed in this paper: (1) refining dense trajectories according to the motion salient region and (2) optimizing the framework of bag-of-words according to the motion salient intensity distribution and the idea of sparse coefficient reconstruction.

In the experiment of refining dense trajectories, the original dense trajectories are extracted using the default parameters in [16], such as *L* = 15, and the dictionary learning in the framework of BOW is using the basic *K*-means clustering algorithm. The selection of the two parameters *λ*_1_ and *λ*_2_ is very important in this paper. In the motion salient map, we define the area as the background or unimportant feature points where the intensity of the feature points is less than *λ*_1_ and define some messy trajectories as the drifting problem that the intensity difference of the feature points between frames is greater than *λ*_2_. Therefore, *λ*_1_ controls the minimum salient intensity of the extracting trajectories region, and *λ*_2_ controls the maximum salient differential intensity between frames of the extracting trajectories region.

**Figure 8:**
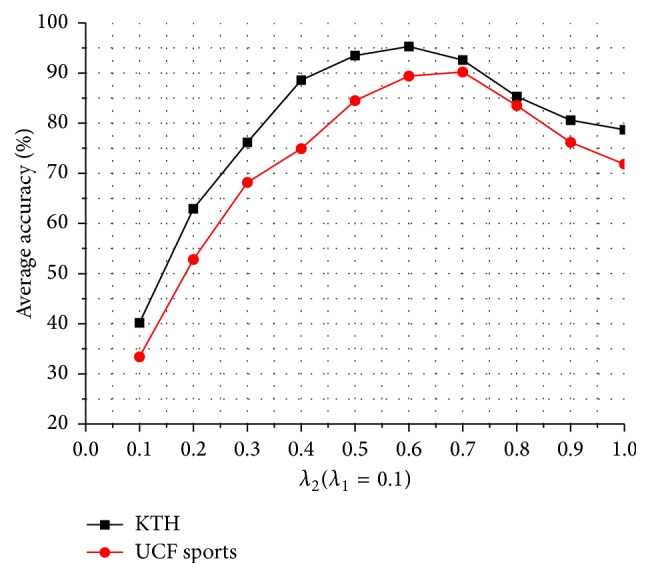
Average accuracy on two action datasets by changing *λ*_2_ and fixing *λ*_1_.

**Figure 9:**
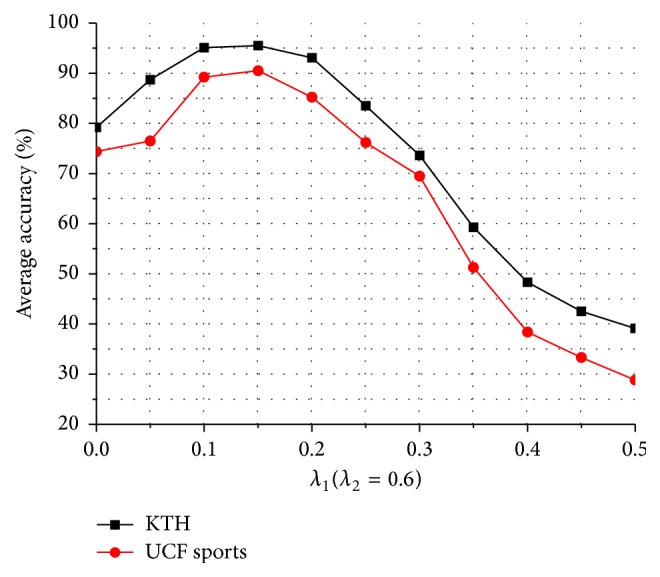
Average accuracy on two action datasets by changing *λ*_1_ and fixing *λ*_2_.

Figure 8 is the curve of the recognition accuracy for KTH and UCF sports datasets when fixing *λ*_1_ and changing *λ*_2_. *λ*_1_ = 0.1 is a predetermined experience value and the intensity of the background region is generally less than 0.1 in the motion salient map extracted in this paper. As the change of *λ*_2_, we can find that it gets a higher recognition accuracy in the two datasets when *λ*_2_ = 0.6.  Figure 9 is the curve of the recognition accuracy for KTH and UCF sports datasets when fixing *λ*_2_ and changing *λ*_1_. As the change of *λ*_1_, we can find that it gets the best effect on removing the background and unimportant feature points and a higher recognition accuracy in the two datasets when *λ*_1_ = 0.15.

According to the experiment above, we use the optimal parameters *λ*_1_ = 0.15 and *λ*_2_ = 0.6 to refine the dense trajectories and compare the recognition accuracy with [16] by the trajectory displacement, HOG, HOF, MBH, and all the combined features.  Table 1 shows that the improved dense trajectories cooperating with a variety of features can improve the recognition accuracy by about 1%.

In the experiment of optimizing the framework of bag-of-words, we use the optimal parameters *λ*_1_ = 0.15 and *λ*_2_ = 0.6 to obtain the final refined dense trajectories and extract the combined features (i.e., trajectory displacement, HOG, HOF, and MBH). Then, we optimize the dictionary learning according to the motion salient intensity distribution and improve the feature representation by referring to the idea of sparse coefficient reconstruction. We compare the improved framework of bag-of-words with the traditional framework of bag-of-words.

**Figure 10:**
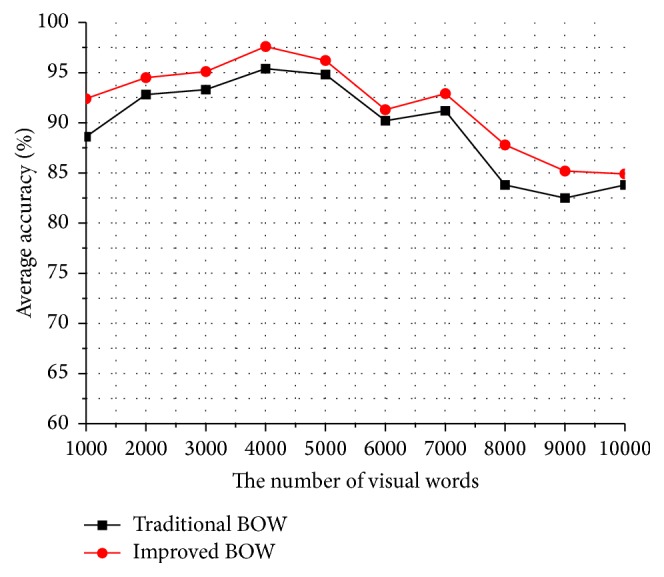
Average accuracy comparison between *K*-means and weighted *K*-means on KTH dataset.

Figure 10 shows the comparing curves of the recognition accuracy on the KTH dataset between the traditional BOW and the improved BOW. We can find that the recognition accuracy of the improved BOW is about 2% higher than the recognition accuracy of the traditional BOW at different number of the visual dictionary. Particularly, when the number of the visual dictionary is smaller, such as 1000, it is not fine enough for feature representation; therefore, the improved BOW has an obvious effect on the recognition accuracy and has improved by over 3%. When the number of the visual dictionary is about 4000, it can get a satisfactory level on the recognition accuracy.

**Figure 11:**
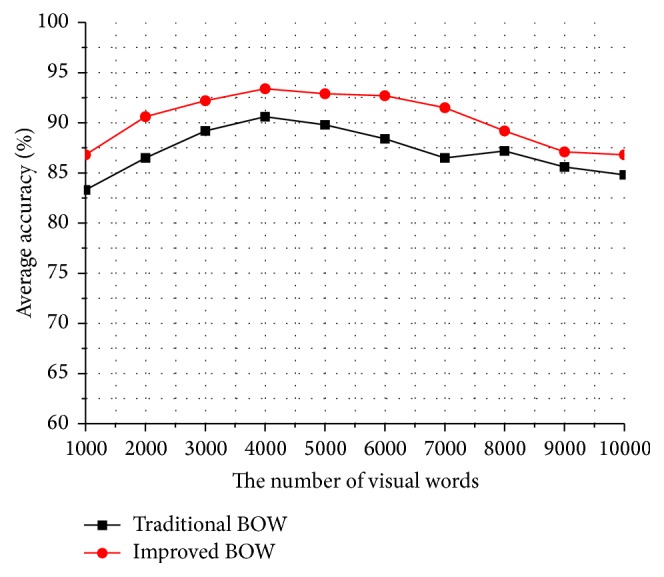
Average accuracy comparison between *K*-means and weighted *K*-means on UCF sports dataset.

Figure 11 shows the comparing curves of the recognition accuracy on the UCF sports dataset between the traditional BOW and the improved BOW. We can find that the improved BOW is basically 3% higher than the traditional BOW at different number of the visual dictionary. As the backgrounds and actions in UCF sports dataset are much complex than those in KTH dataset, the improved BOW can greatly reduce the interference of background and highlight the key motion parts of human body; therefore, the recognition accuracy improvement of UCF sports dataset is more obvious than KTH dataset. Meanwhile, when the number of the visual dictionary is about 4000, both KTH and UCF sports datasets can get the best recognition accuracy.

### 4.4. Comparison of Feature Representation

In this section, we evaluate the performance of the improved salient dense trajectory and the improved BOW model proposed in this paper on the four action datasets of KTH, UCF sports, HMDB51, and UCF50 by using the optimal parameters *λ*_1_ = 0.15 and *λ*_2_ = 0.6 and the visual dictionary number 4000 obtained through the above experiments.

Table 2 shows the comparison of different optimizations based on DTF. DTF stands for the original dense trajectory features [16] and ISDTF and IBOW are the improved salient dense trajectory features and the improved BOW model proposed, respectively, in this paper. We can find that IBOW is around 1% (2%) better than BOW on KTH (UCF sports) for both DTF and ISDTF, and the improvement is much obvious on HMDB51 (UCF50) for about 6% (3%). Furthermore, IBOW + DTF is a bit better than FV + DTF on HMDB51 and is about the same on UCF50.

### 4.5. Comparison to the State-of-the-Art Results

Table 3 is the comparison between our improved human action recognition algorithm and the state-of-the-art experiment results in the recent years. Among them, [16] applies dense trajectory features to describe the human actions and achieve the recognition accuracy of 94.2% and 88.2%, respectively, on KTH and UCF sports datasets. Reference [12] proposes a new spatiotemporal interest point detector based on flow vorticity which can provide prominent spatiotemporal interest points around key positions of the moving foreground and achieve the recognition accuracy of 94% and 88.7%, respectively, on KTH and UCF sports datasets. Reference [9] uses the MBH descriptor based on the differential optical flow to improve the dense trajectory features and gets the improvement of the recognition accuracy; the recognition accuracy on the KTH and UCF sports datasets are 95.3% and 89.1%. Reference [13] presents the novel structure of subspace forest to provide an efficient approximate nearest-neighbor query of subspaces represented as points on Grassmann manifolds; it has a good effect and achieves a recognition accuracy of 97.9% on KTH dataset and 91.3% on UCF sports dataset. These experimental data show that the method based on trajectory is one of the most effective methods in the field of human action recognition.

On the KTH and UCF sports datasets, the recognition accuracy of the improved dense trajectories algorithm proposed in this paper is 97.6% and 93.4%, respectively; it is around 3% higher than the baseline accuracy in [16] and has some advantages comparing with [13].

On the HMDB51 and UCF50 datasets, the recognition accuracy of the improved dense trajectories algorithm proposed in this paper is 56.7% and 90.3%, respectively. It is around 10% (6%) better than the baseline accuracy in [9] on HMDB51 (UCF50). Compared to [29] which estimated camera motion by matching feature points between frames and improved the estimation by a human detector, we use the simple way to eliminate the noise due to background motion, namely, the MBH descriptor. However, our algorithm performs competitively with [29] on both HMDB51 and UCF50.

## 5. Conclusion

In order to improve the effectiveness of the trajectories refinement and feature representation in the human action recognition algorithms based on trajectory, we refine the dense trajectories via the analysis of the motion saliency from the aspect of biology and determine the optimal parameters via experiment. Meanwhile, we optimize the framework of bag-of-words to make the final feature representation more effective according to the motion salient intensity distribution and the idea of sparse coefficient reconstruction. Experimental results show that our algorithm is competitive comparing with the most recent classical algorithms on the four open human action datasets. For the reason that our algorithm only uses a simple way to suppress the camera motion, namely, the MBH descriptor, then other camera motion estimation methods can be considered in the future work. Moreover, our algorithm needs processing each frame of videos and its complexity is high, so it is difficult to achieve the real-time processing on the existing hardware. The future work will mainly focus on improving the universality and reducing the complexity of the algorithm.

## Figures and Tables

**Table 1:** Average accuracy of dense trajectories and improved dense trajectories on two action datasets.

Method	Features	Average recognition accuracy	
		KTH	UCF sports
Dense trajectories [16]	Trajectory displacement	90.2%	75.2%
	HOG	86.5%	83.8%
	HOF	93.2%	77.6%
	MBH	95.0%	84.8%
	Combined	94.2%	88.2%

Improved DT	Trajectory displacement	92.3%	77.8%
	HOG	86.9%	84.3%
	HOF	94.4%	79.2%
	MBH	93.8%	84.5%
	Combined	95.5%	90.4%

**Table 2:** Comparison of different optimizations based on DTF.

Datasets	BOW		IBOW		Fisher vector (FV)
	DTF	ISDTF	DTF	ISDTF	DTF
KTH	94.2% [16]	95.5%	96.1%	97.6%	—
UCF sports	88.2% [16]	90.4%	90.8%	93.4%	—
HMDB51	46.6% [9]	50.9%	52.9%	56.7%	52.2% [29]
UCF50	84.5% [9]	86.5%	88.5%	90.3%	88.6% [29]

**Table 3:** Comparison to the state-of-the-art results.

KTH	UCF sports	HMDB51	UCF50
DTF + BOW [16] 94.2%	DTF + BOW [16] 88.2%	DTF + BOW [9] 46.6%	DTF + BOW [9] 84.5%
Chen et al. [12] 94%	Chen et al. [12] 88.7%	ITF + FV [29] 57.2%	ITF + FV [29] 91.2%
O'Hara and Draper [13] 97.9%	O'Hara and Draper [13] 91.3%	Stacked FV [30] 56.21%	Shi et al. [31] 83.3%
ISDT + IBOW (ours) 97.6%	ISDT + IBOW (ours) 93.4%	ISDT + IBOW (ours) 56.7%	ISDT + IBOW (ours) 90.3%
